# Richness and Diversity of Phlebotomine Sand Flies (Diptera: Psychodidae) in North Khorasan Province, Northeast of Iran

**Published:** 2018-09-30

**Authors:** Kourosh Arzamani, Hassan Vatandoost, Yavar Rassi, Amir Ahmad Akhavan, Mohammad Reza Abai, Mohammad Alavinia, Kamran Akbarzadeh, Mehdi Mohebali, Sayena Rafizadeh

**Affiliations:** 1 Department of Medical Entomology and Vector Control, School of Public Health, Tehran University of Medical Sciences, Tehran, Iran; 2 Vector-Borne Diseases Research Center, North Khorasan University of Medical Sciences, Bojnurd, Iran; 3 Department of Environmental Chemical Pollutants and Pesticides, Institute for Environmental Research, Tehran University of Medical Sciences, Tehran, Iran; 4 Toronto Rehabilitation Centre, University Health Network, Toronto, Canada; 5 Department of Medical Parasitology, School of Public Health, Tehran University of Medical Sciences, Tehran, Iran; 6 Ministry of Health and Medical Education, National Institute for Medical Research Development, Tehran, Iran

**Keywords:** Richness, Diversity, Sandflies, *Leishmania*, Iran

## Abstract

**Background:**

We aimed to determine the species composition, richness and diversity indices of the Phlebotomine sand flies at different topographic condition in visceral (VL) and cutaneous leishmaniasis (CL) foci in the northeast of Iran.

**Methods:**

This cross-sectional study was conducted during 2016 in North Khorasan Province. The sampling was focused on rural regions, where human cases of VL and/or CL were diagnosed and reported during last 5 years. Sand flies were collected three times each twenty days during peak periods of seasonal activity. Seven collection methods were used. Some Alpha and Beta diversity indices were calculated.

**Results:**

Overall, 7253 sand flies were collected and identified. They were from 19 species of Phlebotominae sand-flies. *Phlebotomus sergenti* and *Ph. papatasi* were the most prevalent (84.9%) species in the study area. Species richness (S) was very different in three areas and were18, 8, and 4 respectively but Evenness (E) were 0.357, 0.345, and 0.380, so evenness was almost equal in the study areas. Shannon Index (H) and Margalef Richness Index were calculated 1.033, 0.718, 0.527 and 2.117, 0.8998, 0.4006 respectively.

**Conclusion:**

The sand fly fauna in North Khorasan Province was very rich and often included some of the most important proven or suspected vectors of leishmaniasis. Species diversity indices (Shannon index, and Simpson’s index) were not high due to decreasing in evenness. The Margalef richness index could accurately reflect the biodiversity of sand flies between three subtidal locations.

## Introduction

Phlebotominae sandflies (Diptera: Psychodidae: Phlebotominae) are widespread in the tropical and subtropical regions. They may carry and transmit etiologic agents of arbo-viruses and bartonellosis but the most important agents that they transmit are *Leishmania*, protozoan parasites (1). There are three main forms of leishmaniases, visceral leishmaniasis (VL), various forms of cutaneous leishmaniasis (CL), and mucocutaneous leishmaniasis. Endemic leishmaniasis transmission has been reported from at least 98 countries and three territories on five continents. Cutaneous leishmaniasis is more widely distributed and approximately 0.7 to 1.2 million new cases occur each year. Ten countries with highest estimated CL cases are Afghanistan, Algeria, Colombia, Brazil, Iran, Syria, Ethiopia, North Sudan, Costa Rica and Peru, together account for 70% to 75% of global estimated CL incidence (2).

Approximately 800 species of phlebotomine sand flies are distributed in the world. In Iran, 47 species of sand flies have been identified and the presences of some species are doubtful (3–5). CL is reported from all provinces and is endemic in more than half of the 31 provinces in Iran. VL is endemic in northwest, west, southwest, central and north-east of Iran and is reported sporadically from all provinces of the country (5–7).

The first entomological studies in North Khorasan Province were conducted during 1975 in Esfarayen County and *L. major* detected in *Phlebotomus papatasi* (8). In Shirvan County molecular infection of leishmanial due to *L. infantum* was observed in *P. kandelakii* (9). Cutaneous leishmaniasis is reported from all counties and VL is reported from five out of eight counties of North Khorasan Province (10). Some counties in Northern half of the province have been reported to be endemic for VL with more than 160 new cases in the last second decade (11).

Biodiversity is a primary interest of ecologists and is a contraction of biological diversity. Species, ecosystem, and genetic diversity are three components of biodiversity. Diversity can be represented in various types of Alpha (α), Beta (β) and Gamma (γ). Alpha diversity is the variation of species within a community or habitat, Beta diversity is in taxonomic composition between communities, and Gamma diversity is the total variation of an area and combination of both alpha and beta diversity (12).

Species diversity is a central theme in ecology and has two separate components named species richness (S) and evenness (E). Richness and evenness have been combined mathematically in various ways to calculate diversity indices based on proportional abundances of species (12). Two indices of Shannon–Wiener (Shannon index) and Simpson’s index commonly used for species biodiversity. Simpson’s index measures the probability of two individuals randomly selected from a sample belongs to the same species. Shannon index is one of the most well-known diversity indices and measures species richness and proportion of each species within a community (12, 13).

The two indices differ in their emphasis on species richness (Shannon–Wiener) or abundance (Simpson’s). Typical values of Shannon–Wiener index are between 1.5 and 3.5 and rarely go beyond to 4.5 (12).

Diversity also can be measured as the variation in species composition among communities (Beta diversity). Several techniques have been developed to compare communities based on their species compositions. The Jaccard’s similarity coefficient (CJ) is the simplest of these similarity measures. This index will always give a value between 0 and 1 and then multiply by 100 to be expressed as percentage of similarity (12).

Knowledge about the ecological aspects of sand fly species and biodiversity indices help in understanding the transmission dynamics of the disease to human and reservoirs.

The purpose of this study was to determine the species composition, richness and diversity indices of the Phlebotomine sand flies at different topographic condition e.g. mountain/plain in North Khorasan Province located in the northeast of Iran.

## Materials and Methods

### Study area

This cross-sectional study was conducted during 2016 in some rural areas of North Khorasan Province, between 36°37′–38°17′ N latitudes and 55°53′–58°20′ E longitudes. The province has a desert, mountainous and temperate climate with cold winters and is bordered by Turkmenistan in the north. The total area is approximately 28434km^2^ and situated in the northeast of Iran. Bojnurd is the capital city of the province. The Koppeh Dagh Mountain, mountain range on the border of Turkmenistan and Iran and The Eastern Alborz range located in this province.

### Selection of the villages of study

**Fig. 1.**
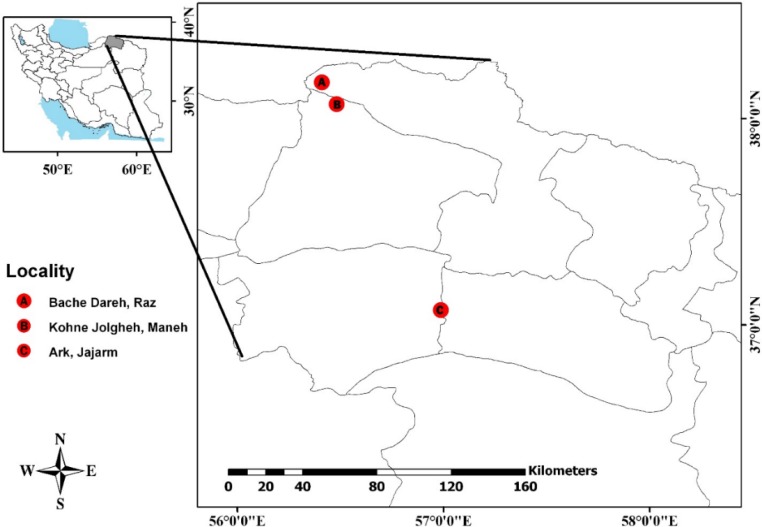
Study area and geographical locations of phlebotomine sand flies, North Khorasan Province, Iran, 2016

The sampling was focused on rural regions. Three villages including A) Bacheh-Dareh located in Raz and Jargalan County, B) Kohne Jolgeh located in Maneh and Samalqan County and C) Arg located in Jajarm County were selected based on diagnosed human VL and/or CL cases during last 5 years. Collection site in Bacheh-Dareh was a valley 3km far from the village and sand fly captures have been restricted to outdoor and wild environments. Collection sites in Kohne Jolgeh were indoors and domestic environments and in Arg were indoors and outdoors and domestic environments. Several human VL and CL had been reported from sites of A and B but only human CL cases had been reported from site of C (Fig. 1).

### Sand fly collection

Sand flies were collected three times each twenty days during the peak activity periods of sand flies. Sample collection started from early July and continued until late Aug 2016. Seven collection methods were used as follows: 1) Sticky paper Traps (SPT). We installed 10 paper traps for each rotation and totally 60 papers per night. 2) Light Trap (LTP), and 3) CDC light trap baited with carbon dioxide (CO_2_) gas that obtained by a CO_2_ gas tank (CLT). The light traps were suspended at 1.5m above the ground. 4) White Shannon Trap (WST), 5) Black Shannon Trap (BST) were made of white or black cloth and consisted of a large central compartment and two smaller lateral ones. The measurements, in meters (width, length, and height), of the central and the lateral compartments were 1.3× 1.3× 2m respectively and suspended by cords from supports and base of the traps touched the ground. A portable stove was used as CO_2_ and light source inside the traps. 6) Animal Baited Trap (ABT) and finally 7) Disney Trap (DST). All of the traps were placed randomly with a distance of 20m from each other. The traps were set before sunset, were changed every two hours and remained in operation during the night (20:00 to 08:00). As the traps were changed the new ones were replaced in the same location. Traps were rotated clockwise between the trap locations in site “A” and” C” but were fix in site “B”. Collected sand flies were stored in 96% ethanol alcohol. The specimens were mounted on glass slides in Puri’s medium. Species identification was carried out according to morphological characters using pictorial keys of sand flies (14).

### Determination of the species diversity

Species diversity based on Simpson index and Shannon-Wiener index, species richness based on Margalef index and Menhinick index and also Evenness based on Shannon-Wiener evenness index and Buzas and Gibson’s evenness index have been calculated to estimate species biodiversity of Phlebotomine sand flies in the study area. Computation of these indices is shown in (Table 1).

**Table 1.** Different diversity indices of sand flies of the collection sites, North Khorasan Province, Iran, 2016

**Index**	**Computation**	**Collection sites**		

		**A**	**B**	**C**
**specimens**	Total number of specimens	3073	2392	1788
**Species richness (S)**	The number of species	18	8	4
**Shannon Index (H ′)**	H'=∑i=1spi ln⁡ (pi)	1.033	0.718	0.527
**Simpson Index**	$\frac{\sum_{i=1}^{s}ni\;(ni-1)}{N(N-1)}$	0.6185	0.6468	0.6865
**Shannon Evenness Index (E)**	SHEI= H/ln (S)	0.357	0.345	0.380
**Buzas and Gibson’s Evenness Index**	$\frac{e-\sum_{i=1}^{s}pi\;\ln\;(pi)}{S}$	0.1561	0.2562	0.4235
**Berger-Parker Dominance Index**	$\frac{n\;\max}{\text{N}}$	0.7829	0.7889	0.8082
**Menhinick Richness Index**	$\frac{S}{\sqrt{\sum_{i=1}^{s}ni}}$	0.3247	0.1636	0.0946
**Margalef Richness Index**	$\frac{S-1}{\ln\;N}$	2.117	0.8998	0.4006

(A: Bacheh-Dareh village, B: Kohne Jolgeh village, C: Arg village)

Beta diversity was also estimated by similarity between different communities using Jaccard’s similarity coefficient. Berger-Parker dominance index and Lorenz graph of the collected sand flies within different collection sites were estimated. The statistical analyses were performed in SPSS version 18 (Chicago, IL, USA).

## Results

An overall 7253 sand flies (19 species) from three mentioned villages were collected and identified. Ten species belonged to the phlebotomus genus and nine species belong to the genus Sergentomia. The overall numbers of collected females were 3543 and consisted of 48.8% of specimens. *Phlebotomus sergenti* and *Ph. papatasi* were the most prevalent (84.9%) species in the study area. *Phlebotomus sergenti* was the most predominant species being recorded in all collected areas. Species composition and relative abundance of collected phlebotomine sand flies is shown in (Table 2).

**Table 2.** Species composition and relative abundance of phlebotomine sand flies in North Khorasan Province, Iran, 2016

**Species**	**Collection sites**			**Total**	**%**

	**A**	**B**	**C**		
***Ph. sergenti***	2406	1887	15	4308	59.4
***Ph. papatasi***	42	366	1445	1853	25.5
***Ph. alexandri***	137	76	0	213	2.9
***Ph. major***	52	9	0	61	0.8
***Ph. caucasicus***	6	2	0	8	0.1
***Ph. mongolensis***	9	0	0	9	0.1
***Ph. halepensis***	30	0	0	30	0.4
***Ph. longiductus***	5	0	0	5	0.1
***Ph. turanicus***	3	0	0	3	0.0
***Ph. ansarii***	0	0	1	1	0.0
***Se. sintoni***	4	0	327	331	4.6
***Se. sumbarica***	117	18	0	135	1.9
***Se. grekovi***	4	0	0	4	0.1
***Se. dentata***	19	14	0	33	0.5
***Se. theodori***	67	0	0	67	0.9
***Se. hodgsoni***	41	0	0	41	0.6
***Se. pawlowskyi***	72	0	0	72	1.0
***Se. dreyfussi turkestanica***	54	20	0	74	1.0
***Se. clydei***	5	0	0	5	0.1
**Total**	3073	2392	1788	7253	100.0

(A: Bacheh-Dareh village, B: Kohne Jolgeh village, C: Arg village)

**Fig. 2.**
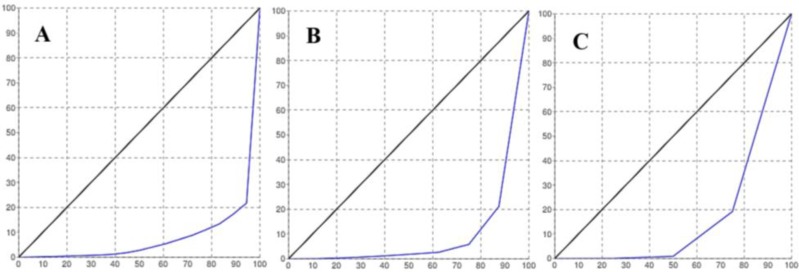
The Lorenz graph for phlebotomine sand flies collected in different collection sites, North Khorasan Province, Iran, 2016. (A: Bacheh-Dareh village, B: Kohne Jolgeh village, C: Arg village)

Some of the most important Alpha diversity indices including, Species richness, Shannon Index, Simpson index, Buzas and Gibson’s evenness index, Berger-Parker dominance index, Menhinick richness index, and Margalef richness index were calculated and are shown in Table 2. The Lorenzen curve is shown the cumulative percentage in relation to species rank or log species rank (Fig. 2).

The sand fly community similarity measured by Jacard’s similarity coefficient. The similarity of the sand fly communities was the highest (42.1%) between community A and B and was the lowest (15.8%) between community A and C. Jacard’s coefficient was 20% between community B and C.

## Discussion

This study reports some proven or suspected vectors of CL and VL in Iran including *Ph. papatasi*, *Ph. sergenti*, *Ph. caucasicus*, *Ph. alexandri* and *Ph. major*. Among the collected species presence of *Sergentomyia dreyfussi turkestanica* in Iran was doubtful and we collected and confirmed the presence of this species in the country. *Phlebotomus turanicus* was reported from this region (3) and we collected and confirmed the presence of this species in Iran. *Phlebotomus kandelakii* had been reported as the probable vector of VL in North Khorasan Province (9) but we were not able to capture in this investigation.

*Phlebotomus sergenti* was the most predominant species throughout the study area and recorded in all localities. Our results are similar to another study that reported *Ph. sergenti* has a wide distribution in the country and includes and extends beyond the distribution of *L. tropica* (5).

*Phlebotomus papatasi* normally prefers to live in plain areas rather than in mountains. This species often abundant in areas of steppe and semi-arid zones where temperatures are high and such circumstance appears in community C. *Phlebotomus papatasi* showed a widespread distribution in this region and in community C about 81% of all specimens belong to this species. Low frequency of this species in mountains area such as community A could be explained by its preference to semi-arid areas (15, 16).

*Phlebotomus alexandri* and *Ph. major* were found in A and B collection sites recognized as the areas of the richest biodiversity of sandflies. *Phlebotomus major* has been reported from 17 out of 31 provinces and in all areas which human cases of ZVL have been reported mostly in mountainous areas (5). *Phlebotomus alexandri* has been reported as a probable vector of zoonotic visceral leishmaniasis (ZVL) in Iran (17) and is generally distributed in mountainous regions although reported from almost all parts of Iran (5). These species were reported from different areas of Iran including plain and highlands. *Phlebotomus alexandri* prefers regions with a high percentage of relative humidity and warmer niches (16). Caspian Sea near of these villages provides relative humidity suitable for the presence of these species.

In the study area, nineteen species of sand flies collected and identified. This revealed sand flies species richness in this area was higher than some other regions in the country (15, 18, 19).

There were differences in species richness and diversity indices in three communities. In community A, the Shannon diversity index and richness were maximum (H’= 1.033, S= 18) and more than community B and C. There are only limited comprehensive studies on biodiversity of sand flies in Iran in which the authors calculated the relevant indices (15, 18–20). In a study in Qom Province, Shannon diversity index in mountainous areas (H’= 1.36, S= 9) was higher than in lowlands (H= 0.66, S= 9). The highest diversity in community A is similar to this investigation that showed more diversity in the mountainous area (15, 19), but in contrast to evenness. The evenness in Qom Province in the mountainous areas was 0.62, and in lowlands was 0.30, while in all communities in North Khorasan Province were similar (from E= 0.345 up to 0.380) and less than mountainous areas in Qom Province.

Species diversity, as indicated by the values of Shannon-Wiener index (H) consists of two components: species richness and evenness. A community is said to have high species diversity if many species are present and all species are nearly equally abundant but in all communities in the study area one or two species (*Ph. sergenti* and *Ph. papatasi*) consist of more than 80 percent of specimens as seen in table of species composition (Table. 1), Berger-Parker Dominance Index (Table 2) and Lorenz graph (Fig. 2) and therefore, evenness is less than other studies and species diversity is not much.

The similarity of the sand fly communities was different based on Jacard’s similarity coefficient. The similarity of the sand fly communities was highest (from 40% up to 100%) between communities (18). The results were in contrast to our finding and showed more diversity of the sand fly communities in the North Khorasan Province. Alborz Mountain range and Aladagh Mountain in the middle parts of the province appear to be important geographical barriers for sand fly distribution. Future investigations are needed to clarify the role of natural barriers such as mountains for sand fly distribution.

## Conclusion

The sand fly fauna in North Khorasan Province was very rich and often included some of the most important proven or suspected vectors of leishmaniasis. Species diversity indices (Shannon index, and Simpson’s index) were not high due to decreasing in evenness. Some well-known species from mountainous areas such as *Ph. alexandri*, *Ph. major*, *Ph. halepensis*, *Ph. longiductus* and *Ph. turanicus* and plain area species such as *Ph. papatasi* and *Ph. caucasicus* were collected that shows the potential of transmission of a different kind of leishmaniasis in this province.

The species richness of sand flies was varied between three selected leishmaniasis foci in the North Khorasan Province and the Margalef richness index could accurately reflect the biodiversity of sand flies between three collection areas.

## References

[1] Maroli M Feliciangeli M Bichaud L Charrel R Gradoni L (2013) Phlebotomine sandflies and the spreading of leishmaniases and other diseases of public health concern. Med Vet Entomol. 27 (2): 123–47. 22924419 10.1111/j.1365-2915.2012.01034.x

[2] Alvar J Vélez ID Bern C Herrero M Desjeux P Cano J Jannin J den Boer M WHO Leishmaniasis Control Team (2012) Leishmaniasis worldwide and global estimates of its incidence. PloS One. 7(5): e35671. 22693548 10.1371/journal.pone.0035671 PMC3365071

[3] Akhoundi M Parvizi P Baghaei A Depaquit J (2012) The subgenus *Adlerius* Nitzulescu (Diptera: Psychodidae, *Phlebotomus*) in Iran. Acta Trop. 122 (1): 7–15. 22079375 10.1016/j.actatropica.2011.10.012

[4] Zahraei-Ramazani AR Kumar D Yaghoobi-Ershadi MR Naghian A Jafari R Shirzadi MR Abdoli H Soleimani H Shareghi N Ghanei M Arandian MH Hanafi-Bojd AA (2013) Sand flies of the subgenus *Adlerius* (Diptera: Psychodidae) in an endemic focus of visceral leishmaniasis and introduction of *Phlebotomus* (*Adlerius*) *comatus* as a new record for Iran. J Arthropod-Borne Dis. 7(1): 1–7. 23785689 PMC3684492

[5] Yaghoobi-Ershadi MR (2012) Phlebotomine sand flies (Diptera: Psychodidae) in Iran and their role on *Leishmania* transmission. J Arthropod Borne Dis. 6(1): 1–17. 23293774 PMC3528173

[6] Mohebali M (2013) Visceral leishmaniasis in Iran: review of the epidemiological and clinical features. Iran J Parasitol. 8(3): 348–358. 24454426 PMC3887234

[7] Karimi A Hanafi-Bojd AA Yaghoobi-Ershadi MR Akhavan AA Ghezelbash Z (2014) Spatial and temporal distributions of phlebotomine sand flies (Diptera: Psychodidae), vectors of leishmaniasis, in Iran. Acta Trop. 132: 131–139. 24462940 10.1016/j.actatropica.2014.01.004

[8] Javadian E Nadim A Tahvildare-Bidruni G Assefi V (1976) Epidemiology of cutaneous leishmaniasis in Iran: B. Khorassan Part V: Report on a focus of zoonotic cutaneous leishmaniasis in Esferayen. Bull Soc Pathol Exot Filiales. 69(2): 140–143. 1037090

[9] Rassi Y Abai M Oshaghi M Javadian E Sanei A Rafidzadeh S Arzamani K (2012) First detection of *Leishmania infantum* in *Phlebotomus kandelakii* using molecular methods in north-eastern Islamic Republic of Iran. East Mediterr Health J. 18(4): 387–92. 22768703 10.26719/2012.18.4.387

[10] Rajabzadeh R Arzamani K Shoraka H Riyhani H Hosseini SH (2015) Epidemiological survey and geographical distribution of cutaneous Leishmaniasis in North Khorasan Province, 2006–2013. Int J Epid Res. 2(4): 197–203.

[11] Arzamani K (2012) Visceral leishmaniasis in North Khorasan Province, north east of Iran. Int J Infect Dis. 16: e340–341.

[12] Magurran A (2004) Measuring biological diversity. Blackwells Oxford, UK.

[13] Schowalter TD (2016) Insect ecology: an ecosystem approach. Academic Press.

[14] Rassi Y Hanafi-Bojd AA (2006) Phlebotomine sand flies, vectors of Leishmaniases: Morphology, biology, ecology, and field and laboratory methods with pictorial key of Iranian sand flies. Noavaran-Elm Publication Tehran, Iran.

[15] Jahanifard E Yaghoobi-Ershadi MR Akhavan AA Akbarzadeh K Hanafi-Bojd AA Rassi Y Sedaghat MM Shirzadi MR Karimi A (2014) Diversity of sand flies (Diptera, Psychodidae) in southwest Iran with emphasis on synanthropy of *Phlebotomus papatasi* and *Phlebotomus alexandri*. Acta Trop. 140: 173–180. 25159535 10.1016/j.actatropica.2014.08.017

[16] Simsek FM Alten B Caglar SS Ozbel Y Aytekin AM Kaynas S Belen A Kasap OE Yaman M Rastgeldi S (2007) Distribution and altitudinal structuring of phlebotomine sand flies (Diptera: Psychodidae) in southern Anatolia, Turkey: their relation to human cutaneous leishmaniasis. J Vector Ecol. 32(2): 269–279. 18260517 10.3376/1081-1710(2007)32[269:daasop]2.0.co;2

[17] Azizi K Rassi Y Javadian E Motazedian M Rafizadeh S Yaghoobi-Ershadi MR Mohebali M (2006) *Phlebotomus* (*Paraphlebotomus*) *alexandri*: a probable vector of *Leishmania infantum* in Iran. Ann Trop Med Parasitol. 100(1): 63–68. 16417715 10.1179/136485906X78454

[18] Akhoundi M Mirzaei A Baghaei A Alten B Depaquit J (2013) Sand fly (Diptera: Psychodidae) distribution in the endemic and non-endemic foci of visceral leishmaniasis in northwestern Iran. J Vector Ecol. 38(1): 97–104. 23701613 10.1111/j.1948-7134.2013.12014.x

[19] Abedi-Astaneh F Akhavan AA Shirzadi MR Rassi Y Yaghoobi-Ershadi MR Hanafi-Bojd AA Akbarzadeh K Nafar-Shalamzari R Parsi S Abbasi A Raufi H (2015) Species diversity of sand flies and ecological niche model of *Phlebotomus papatasi* in central Iran. Acta Trop. 149: 246–253. 26071647 10.1016/j.actatropica.2015.05.030

[20] Hazratian T Vatandoost H Oshaghi MA Yaghoobi-Ershadi MR Fallah E Rafizadeh S Shirzadi MR Shayeghi M Akbarzadeh K Rassi Y (2016) Diversity of Sand Flies (Diptera: Psychodidae) in Endemic Focus of Visceral Leishmaniasis in Azar Shahr District, East Azarbaijan Province, North West of Iran. J Arthropod Borne Dis. 10(3): 328–34. 27308291 PMC4906739

